# Atherosclerosis Imaging Quantitative Computed Tomography (AI‐QCT) to guide referral to invasive coronary angiography in the randomized controlled CONSERVE trial

**DOI:** 10.1002/clc.23995

**Published:** 2023-02-27

**Authors:** Yumin Kim, Andrew D. Choi, Anha Telluri, Isabella Lipkin, Andrew J. Bradley, Alfateh Sidahmed, Rebecca Jonas, Daniele Andreini, Ravi Bathina, Andrea Baggiano, Rodrigo Cerci, Eui‐Young Choi, Jung‐Hyun Choi, So‐Yeon Choi, Namsik Chung, Jason Cole, Joon‐Hyung Doh, Sang‐Jin Ha, Ae‐Young Her, Cezary Kepka, Jang‐Young Kim, Jin Won Kim, Sang‐Wook Kim, Woong Kim, Gianluca Pontone, Todd C. Villines, Iksung Cho, Ibrahim Danad, Ran Heo, Sang‐Eun Lee, Ji Hyun Lee, Hyung‐Bok Park, Ji‐min Sung, Tami Crabtree, James P. Earls, James K. Min, Hyuk‐Jae Chang

**Affiliations:** ^1^ The George Washington University School of Medicine Washington District of Columbia USA; ^2^ Jefferson Medical Institute Philadelphia Pennsylvania USA; ^3^ Centro Cardiologico Monzino IRCCS Milan Italy; ^4^ CARE Hospital and FACTS Foundation Hyderabad India; ^5^ Quanta Diagnostico Nuclear Curitiba Brazil; ^6^ Gangnam Severance Hospital Seoul South Korea; ^7^ Pusan National University Hospital Busan South Korea; ^8^ Ajou University Hospital Gyeonggi‐do South Korea; ^9^ Severance Cardiovascular Hospital, Yonsei University Health System Seoul South Korea; ^10^ Cardiology Associates of Mobile Mobile Alabama USA; ^11^ Inje University, Ilsan Paik Hospital Gyeonggi‐do South Korea; ^12^ Gangneung Asan Hospital Gangwon‐do South Korea; ^13^ Kangwon National University Hospital Gangwon‐do South Korea; ^14^ National Institute of Cardiology Warsaw Poland; ^15^ Wonju Severance Hospital Gangwon‐do South Korea; ^16^ Korea University Guro Hospital Seoul South Korea; ^17^ Chung‐Ang University Hospital Seoul South Korea; ^18^ Yeungnam University Hospital Daegu South Korea; ^19^ University of Virginia Medical Center Charlottesville Virginia USA; ^20^ VU Medical Center Amsterdam the Netherlands; ^21^ Hanyang University, Hanyang University Medical Center Seoul South Korea; ^22^ Myongji Hospital, Seonam University College of Medicine Gyeonggi‐do South Korea; ^23^ International St. Mary's Hospital, Catholic Kwandong University College of Medicine Incheon South Korea; ^24^ Cleerly Inc New York New York USA

**Keywords:** artificial Intelligence, atherosclerosis, CCTA, coronary artery disease, coronary computed tomography, fractional flow reserve, quantitative coronary angiography

## Abstract

**Aims:**

We compared diagnostic performance, costs, and association with major adverse cardiovascular events (MACE) of clinical coronary computed tomography angiography (CCTA) interpretation versus semiautomated approach that use artificial intelligence and machine learning for atherosclerosis imaging‐quantitative computed tomography (AI‐QCT) for patients being referred for nonemergent invasive coronary angiography (ICA).

**Methods:**

CCTA data from individuals enrolled into the randomized controlled Computed Tomographic Angiography for Selective Cardiac Catheterization trial for an American College of Cardiology (ACC)/American Heart Association (AHA) guideline indication for ICA were analyzed. Site interpretation of CCTAs were compared to those analyzed by a cloud‐based software (Cleerly, Inc.) that performs AI‐QCT for stenosis determination, coronary vascular measurements and quantification and characterization of atherosclerotic plaque. CCTA interpretation and AI‐QCT guided findings were related to MACE at 1‐year follow‐up.

**Results:**

Seven hundred forty‐seven stable patients (60 ± 12.2 years, 49% women) were included. Using AI‐QCT, 9% of patients had no CAD compared with 34% for clinical CCTA interpretation. Application of AI‐QCT to identify obstructive coronary stenosis at the ≥50% and ≥70% threshold would have reduced ICA by 87% and 95%, respectively. Clinical outcomes for patients without AI‐QCT‐identified obstructive stenosis was excellent; for 78% of patients with maximum stenosis < 50%, no cardiovascular death or acute myocardial infarction occurred. When applying an AI‐QCT referral management approach to avoid ICA in patients with <50% or <70% stenosis, overall costs were reduced by 26% and 34%, respectively.

**Conclusions:**

In stable patients referred for ACC/AHA guideline‐indicated nonemergent ICA, application of artificial intelligence and machine learning for AI‐QCT can significantly reduce ICA rates and costs with no change in 1‐year MACE.

## Key points

- Software platform technology solutions now enable artificial intelligence guided and quantitative cardiac computed tomography angiography (CCTA) for coronary artery plaque quantification and characterization and vascular morphology assessment.
- As most patients referred for invasive coronary angiography (ICA) are found not to have actionable coronary artery disease, artificial intelligence guided CCTA measures of vascular morphology may allow for improved referral management to ICA in a manner that is safe and lower in costs.

## Graphical abstract

Application of artificial intelligence to typically acquired cardiac computed tomography angiography through atherosclerosis imaging and quantitative cardiac computed tomography through the randomized controlled Computed Tomographic Angiography for Selective Cardiac Catheterization Trial is a clinically effective (no change in 1 year major adverse cardiovascular events vs. expert reader), safe (87%−95% reduction in invasive coronary angiography [ICA]) and high value (26%−34% reduction in cost) approach to guide referral management of stable patients being considered for ICA. Clinical case example is shown.

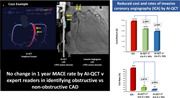

**Abbreviations**
AI: artificial intelligence
AI‐QCT: atherosclerosis imaging and quantitative cardiac computed tomography
CAD: coronary artery disease
CCTA: coronary computed tomography angiography
FDA: food and drug administration
HOPPS: hospital outpatient prospective payment
ICA: invasive coronary angiography
LAD: left anterior descending
LCx: left circumflex
LM: left main
MPI: myocardial perfusion imaging
RCA: right coronary artery

## 1 INTRODUCTION

Invasive coronary angiography (ICA) allows for evaluation of stable symptomatic patients with suspected coronary artery disease (CAD) to guide decisions of coronary revascularization.^1^, ^2^ While current American College of Cardiology (ACC)/American Heart Association (AHA) guidelines outline appropriate selection of patients for elective ICA, in real‐world practice, most individuals who undergo non‐emergent ICA do not have actionable CAD.^3^, ^4^ For these patients, ICA has been shown add to unnecessary health care system costs and increase the risk for potential procedural complications.^5^, ^6^

In the 2021 Updated ACC/AHA Chest Pain guideline, coronary computed tomography angiography (CCTA) has been elevated to a class IA indication to serve as a first line test for identification and exclusion for obstructive CAD with a high sensitivity of 95%−99%.^7^, ^8^, ^9^ Evaluation of CCTA in stable symptomatic patients referred for nonemergent ICA has been done previously in the Coronary Computed Tomographic Angiography for Selective Cardiac Catheterization (CONSERVE) randomized controlled trial (RCT), which observed a selective referral strategy that incorporates a CCTA‐first approach before catheterization was associated with a 77% reduction in ICA.^10^ This deferral of ICA was associated with reduced rates of coronary revascularization and downstream costs, with no differences in 12‐month rates of major adverse cardiovascular events (MACE) as compared to a direct ICA referral strategy.

In this analysis of the CONSERVE RCT, we hypothesized that application of atherosclerosis imaging and quantitative cardiac computed tomography (AI‐QCT) would allow for better determination of patients with and without obstructive CAD who may benefit from ICA, and that this approach would be associated with reduced ICA and lower costs without added risk of MACE.

## 2 METHODS

This study evaluated patients from the Coronary Computed Tomographic Angiography for Selective Cardiac Catheterization (CONSERVE; NCT01810198) RCT who underwent CCTA. For each participant, after receiving written informed consent, eligible patients were randomly assigned to a selective referral or direct referral strategy. This study was a post hoc analysis of the selective referral arm. The original study protocol was approved at each enrolling site by the local institutional review board or ethics committee. Full study details can be found in the landmark publication.^10^ Briefly, CONSERVE was a 1:1 randomized, controlled, open‐label, international, multicenter trial at 22 hospitals and cardiology practices in North America, East Asia, Europe, and India. A selective referral strategy was defined by initial performance of CCTA, with ICA performed at the discretion of the local physician informed by the CCTA findings. The study participants were stable patients with suspected but without known CAD referred for non‐emergent ICA based upon American College of Cardiology/American Heart Association (ACC/AHA) guidelines for ICA, and included indications based on abnormal stress testing or suspected CAD symptoms.^1^, ^11^ Exclusion criteria included known history of CAD, ACC/AHA Class I or III indication for ICA, known complex congenital heart disease, or planned ICA for reasons other than CAD evaluation. Among 784 patients undergoing CCTA in the index study, 37 CCTA studies were not present due to image file corruption. No available CCTA study (0%) was excluded from analysis by AI‐QCT for poor CCTA image quality, with 747 patients included in the final study cohort.

The primary composite endpoint for MACE included death, nonfatal myocardial infarction, unstable angina, stroke, urgent or emergent coronary revascularization, and cardiovascular hospitalization. Further data were collected for downstream invasive and noninvasive coronary procedures, as well as cardiovascular and all cause hospitalizations. The primary endpoint was analyzed at 1 year of follow‐up. Secondary endpoints included evaluation of downstream coronary revascularization, invasive and noninvasive CAD diagnostic testing, and hospitalizations. If a patient had an independent clinical events committee, blinded to randomization assignment, adjudicated all clinical endpoints under the guidance of a Data Safety and Monitoring Board.

CCTA was performed using a single‐ or dual‐source CT scanner with ≥64 detector rows and a detector row width of ≤75 mm in accordance with Society of Cardiovascular Computed Tomography (SCCT) guidelines.^12^ For both ICA and CCTA, presence or absence of angiographic stenoses ≥ 50% and ≥70% was recorded by local site physicians meeting a minimum of Level II or Level III Certification for CCTA interpretation,^13^, ^14^ and the maximum perpatient % stenosis was used to identify the presence or absence of obstructive CAD. Normal ICAs were considered as those that demonstrated no obstructive stenosis ≥ 50% or ≥70%. ICAs was performed in agreement with clinical indications and imaging standards by certified and experienced interventional cardiologists.

**Figure 1:**
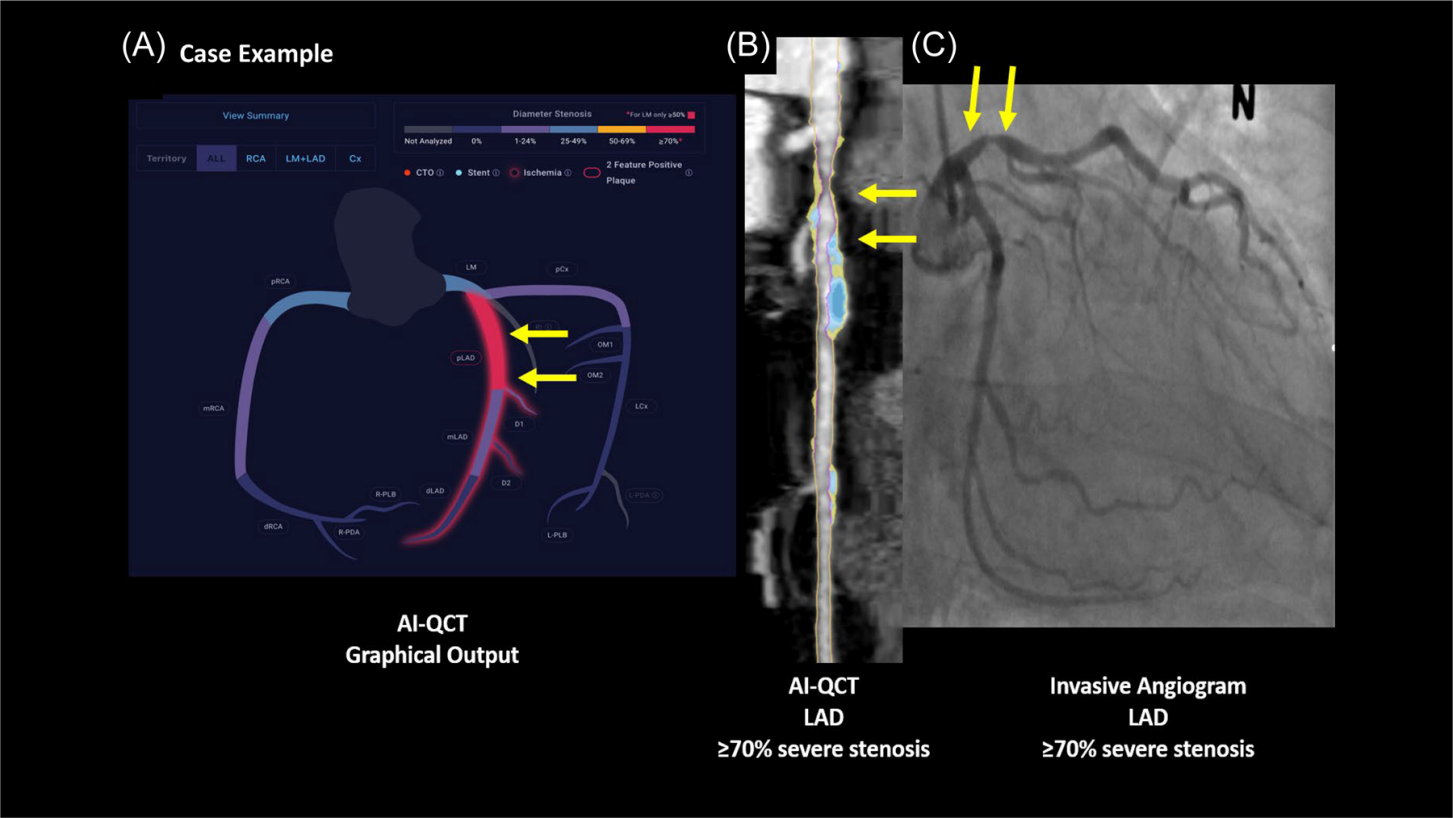
Example of a patient with AI‐QCT analysis demonstrating severe obstructive (>70%) luminal stenosis in the proximal LAD with invasive cath correlation. AI‐QCT total plaque volume, calcified and noncalcified plaque is also shown. AI‐QCT, atherosclerosis imaging and quantitative cardiac computed tomography; LAD, left anterior descending.

AI‐QCT was performed using a commercially available software platform (Cleerly Labs, Cleerly, Inc.) that performs atherosclerosis imaging quantitative CCTA (AI‐QCT) analysis^15^, ^16^, ^17^ using a series of validated convolutional neural network models for quantitative image quality assessment, coronary segmentation and labeling, vascular morphology measurements, and atherosclerotic plaque characterization.^15^ Hundred percent of studies were analyzable by AI‐QCT and included in the study results. A case example with invasive angiography correlation is shown in Figure 1. Prior validation of AI‐QCT has been reported in 2 multicenter trials.^15^, ^17^ Study analysis was performed in‐kind for this investigator‐initiated study.

Coronary segments with a diameter ≥ 2 mm were included in the analysis using a modified 18‐segment SCCT model.^10^, ^16^ Each segment was evaluated for the presence or absence of coronary atherosclerosis, defined as any tissue structure > 1 mm^3^ within the coronary artery wall that was differentiated from the surrounding epicardial tissue, epicardial fat or the vessel lumen itself. The following CAD features were evaluated:

- Stenosis: Utilizing a normal proximal reference vessel cross‐sectional slice, the start and the end of the lesion were identified, and from the cross‐sectional slice that demonstrated the greatest absolute narrowing, % diameter stenosis severity was automatically calculated. Obstructive stenosis was defined at ≥50% and ≥70% stenosis thresholds. All vessels with 0% stenosis were defined as having no CAD.

- Atherosclerosis: Atherosclerosis characterization was performed by AI‐QCT for every coronary artery and its branches. Plaque volumes (PVs) (mm^3^) were calculated for each coronary lesion and then summated to compute the total PV at the patient level. Plaque with a minimum volume of ≥3mm^3^ was included for analysis. This provided data for analysis on both the per‐lesion and per‐patient level. PV was further categorized using Hounsfield unit (HU) ranges with noncalcified plaque (NCP) defined as HU between −30 and +350; low density‐NCP (LD‐NCP) defined as plaques < 30 HU; and calcified plaque (CP) defined as >350 HU.^17^

All statistical analyses were performed using SAS version 9.4 (SAS). Continuous data are reported as mean ± standard deviation, and categorical variables are presented as absolute numbers with corresponding percentages. The rates of stenosis, 0%, 1%−24%, 25%−49%, ≥50% and ≥70% were compared individually between AI‐QCT and Level II/III site readers on a per patient and per vessel basis. The per‐patient differences were evaluated using McNemar's test of the paired data. The per‐vessel rates were compared using the logistic Generalized Estimating Equations method to account for the correlation of the multiple vessels from the same patient. The ability of AI‐QCT and stenosis level II/III site readers to predict the occurrence of a MACE event was compared by generating Receiver Operating Characteristic (ROC) curves for each approach, with stenosis categorized as 0%, 1%−24%, 25%−49%, 50%−69% and 70%−100%. The differences in the predictive ability of each method were compared by calculating and comparing the area under the ROC curves (AUC).

Resource utilization and cost models were established to estimate the rate of downstream ICA using an AI‐QCT‐first approach set at a ≥ 50% and ≥70% to define severe stenosis. The costs of CCTA, invasive angiography and stress testing were determined based on recently published Hospital Outpatient Prospective Payment (HOPPS) standards.^18^ AI‐QCT costs were set at $1500 USD.

## 3 RESULTS

### 3.1 Clinical characteristics of the study population

Demographic and clinical characteristics of the study cohort (60 ± 12 years, 49% women) are listed in Table 1. There was a high prevalence of CAD risk factors, including: 57% hypertension, 33% hyperlipidemia and 30% smokers. 88% of patients experienced symptoms suggestive of CAD, with the majority (70%) having typical or atypical angina.

**Table 1:** Baseline demographics and clinical characteristics.

Variable (% or mean ± SD)	All Patients (*N* = 747)
Age, years	60 ± 12.2
Women	49% (363)
Body mass index, kg/m^2^	25.6 ± 4.0
Race/Ethnicitiy	
African American	0.5% (4)
Asian	86% (639)
Hispanic	0.5% (4)
White	13% (98)
Hypertension	57% (427)
Dyslipidemia	33% (249)
Diabetes	26% (193)
Family history of CAD	9% (67)
Current smoker or history of smoking ≤ 1 year	30% (224)
Symptoms	
Typical angina	30% (224)
Atypical angina	40% (300)
Noncardiac chest pain	2% (17)
Asymptomatic	12% (90)
Other	15% (115)

### 3.2 Comparison of an AI‐QCT approach to clinical CCTA interpretation

Application of AI‐QCT identified 87% and 95% patients without stenosis ≥ 50% and ≥70%, respectively, who would be eligible for ICA deferral (Table 2). For intermediate stenoses 50‐69%, AI‐QCT identified 8% of patients (*n* = 60/747). By comparison, site interpretation by Level II/III readers identified 27% (*n* = 205/747) with ≥50% and 16% (*n* = 117/747) with ≥70% stenosis (*p* < .001), and 12% (*n* = 88/787) patients with intermediate (50%−69%) stenoses who would be eligible for post‐CCTA stress testing after randomization and CCTA.

**Table 2:** Downstream ICA and stress testing after AI‐QCT Applied to CCTA.

Downstream test	Stenosis %	% (Number) by AI‐QCT	% Number by site read (level II/III readers)	*p* Value
ICA (Per vessel)	0%	21% (477/2237)	56% (1253/2237)	<.0001
	1%−24%	55% (1222/2237)	15% (326/2237)	<.0001
	25%−49%	18% (411/2237)	15% (340/2237)	.0113
	≥50%	6% (127/2237)	14% (318/2237)	<.0001
	≥70%	2.1% (47/2237)	7% (163/2237)	<.0001
ICA (Per patient)	0%	9% (67/747)	35% (260/747)	<.0001
	1%−24%	49% (365/747)	16% (117/747)	<.0001
	25%−49%	29% (218/747)	22% (165/747)	<.001
	≥50%	13% (97/747)	28% (208/747)	<.0001
	≥70%	5% (37/747)	16% (117/747)	<.0001
Stress testing (per‐patient)	50%−69%	8% (60/747)	12% (88/747)	<.019

### 3.3 MACE rates

During mean follow‐up of 1.1 ± 0.4 years, 4.3% (*n* = 32) patients experienced MACE (3.8% [n = 29]) for cardiac hospitalization. When stratified by AI‐QCT measures of coronary stenosis (Table 3), amongst the 97 patients with obstructive (≥50%) stenosis, 1 patient (1.0%) suffered cardiovascular death and 1 patient (1.0%) had an acute myocardial infarction. No deaths or myocardial infarctions occurred in 78% (*n* = 583) patients with nonobstructive ≤ 50%. In addition, for nonobstructive ≤ 50%. patients (*n* = 583), 1 (1.5%) patient by AI‐QCT 0% stenosis had a cardiac hospitalization. 24 (4.1%) had MACE excluding cardiovascular death or acute myocardial infarction including unstable angina (6, 1.0%), cardiac hospitalization (22, 3.8%) and/or stroke (2, 0.3%). When categorizing stenosis severity as 0%, 1%−24%, 26%−49%, 50%−69%, >70%, stenosis severity to predict MACE events was similar between AI‐QCT (AUC of 0.61; 95% CI 0.52−0.70) and Level II/III CCTA interpretation (AUC of 0.63; 95% CI 0.53−0.73; *p* = .64). AI‐QCT‐based quantification of atherosclerotic plaque demonstrated a linear and significant association between the absolute PV and MACE with a hazard ratio for each PV category of 2.0 (95% CI 1.3−3.0; *p* = .0012). For patients with PV between 0 and 300 mm^3^ (*n* = 509), 301−750 mm^3^ (*n* = 174) and ≥750 mm^3^ (*n* = 64), there was an observed MACE rate of 2.6%, 7.0%, and 9.4%, respectively, (*p* = .001).

**Table 3:** MACE rate by AI‐QCT stenosis measurements.

MACE Endpoints	ALL (*n* = 747)	0% (*N* = 67)	1%−49% (*N* = 583)	≥50% (*N* = 97)	≥70% (*N* = 37)
CV Death	1 (0.1%)	0 (0%)	0	1 (1.0%)	0 (0%)
Acute myocardial infarction	1 (0.1%)	0 (0%)	0 (0%)	1 (1.0%)	0 (0%)
Unstable angina	6 (0.8%)	0 (0%)	6 (1.0%)	0 (0%)	0 (0%)
Cardiac hospitalization	29 (3.8%)	1 (1.5%)	22 (3.8%)	5 (5.2%)	1 (2.7%)
Stroke	2 (0.3%)	0 (0%)	2 (0.3%)	0 (0%)	0 (0%)

### 3.4 Cost‐analysis

Results of an AI‐QCT‐based strategy for referral management of only patients with high‐grade stenosis to ICA are listed in Table 4. At *a* ≥ 50% and ≥70% stenosis threshold, application of AI‐QCT would have resulted in 87% and 95% patients, respectively, avoiding unnecessary ICA at a 26% and 34% cost‐savings, respectively.

**Table 4:** Diagnostic cost of strategies for direct ICA referral and referral to ICA based upon AI‐QCT.

Scenario	*N*	Cost	Cost/Patient	Change
Straight to ICA				
ICA	784	$2, 175, 600	$2775	
AI‐QCT, followed by ICA if ≥ 50%				
CCTA	747	$135, 954		
AI‐QCT	747	$1, 120, 500		
ICA	97	$269, 175		
Total		$1, 525, 629	$2042	‐26%
AI‐QCT, followed by ICA if ≥ 70%				
CCTA	747	$135 954		
AI‐QCT	747	$1 120 500		
ICA	37	$102 675		
Total		$1 359 129	$1819	‐34%

## 4 DISCUSSION

In this present study, we evaluated for the first time an AI‐QCT strategy to guide judicious referral to nonemergent ICA for patients with an ACC/AHA Guideline indication and determined that adoption of an AI‐QCT approach could reduce unnecessary ICA by 87%−95% based upon stenosis severity thresholds. The rates of safe ICA deferral from AI‐QCT were significantly higher than those based upon Level II/III reader interpretation of CCTA. Further, the AI‐QCT approach was safe, with no patient experiencing MACE during the length of the follow‐up period who had been quantified as having non‐severe stenosis by AI‐QCT. Finally, an AI‐QCT approach was cost‐efficient compared to standard of care Level II/III CCTA interpretation, with a 26‐34% reduction in costs by AI‐QCT‐based ICA deferral.

To our knowledge, these present study results represent the first to evaluate within a multicenter RCT the clinic‐economic feasibility of an AI‐QCT approach for comprehensive assessment of atherosclerosis, stenosis and other vascular morphology features for determining appropriateness of ICA for patients with guideline indications for nonemergent catheterization. Our findings provide strong evidence that integration of leading‐edge machine intelligence tools applied to CCTA can have large implications in the proper selection of patients for ICA versus those who can safely avoid unnecessary invasive, expensive, and potentially harmful procedures. The additional prognostic utility of quantified atherosclerotic burden by AI‐QCT for robust identification of individuals at risk of future MACE, as was observed in this study, provides significant incremental value for the widespread use of AI‐QCT in clinical practice.

Our study results are in direct accordance with recent data published from RCTs have established a utility of CCTA to guide decisions of ICA referral. As an example, in the Prospective Multicenter Imaging Study for Evaluation of Chest Pain (PROMISE) Trial, use of CCTA was associated with fewer catheterizations showing no obstructive CAD than was functional testing (3.4% vs. 4.3%, *p* = .02).^19^ In a comparison to expert core laboratory, clinical site readers demonstrated significant overestimation of stenosis, with a 68% increased erroneous rate of severe stenosis. This overestimation may have influenced the higher rates of ICA in study, and is keeping with our current findings wherein clinical CCTA interpretation was associated with a significantly higher rate of false positive severe stenoses compared to a validated AI‐QCT platform.

The importance of these findings stems from prior information reported from the National Cardiovascular Data Registry which demonstrated that nearly 2/3 patients referred for ICA will not, in fact, be found to have actionable CAD.^4^ While some of this ICA normalcy may be attributed to inappropriate referral for non‐guideline‐indicated reasons, the current study restricted enrollment to those patients with specific ACC/AHA recommended indication, and still identified the majority of patients to not, in fact, have any stenosis ≥ 50% or ≥70%. These data have important ramifications not only to the use of ICA as a diagnostic modality but also percutaneous coronary intervention (PCI) as a therapeutic modality. Prior studies have exhibited a strong relationship between ICA and PCI, particularly when they are performed at the same setting.^20^ This so‐called “diagnostic‐therapeutic cascade,” if broken, may reduce unnecessary PCI for patients who will not benefit from its performance.^21^ In the original CONSERVE trial, PCI rates were reduced by ~50% and, based upon the current study findings, could be further reduced by application of an AI‐QCT strategy.

## 5 LIMITATIONS

The present study is not without limitations. The current analyses were performed in post hoc fashion from an international, multicenter, RCT. Further, AI‐QCT was compared to clinical site interpretation by expert readers, but no blinded CCTA core laboratory was employed. Similarly, as the CONSERVE trial evaluation of ICA was done in pragmatic fashion, no blinded quantitative coronary angiography (QCA) analysis was performed and AI‐QCT could not be directly compared to QCA for diagnostic accuracy measures. However, in prior multicenter clinical trials, AI‐QCT has been previously demonstrated as having robust diagnostic performance compared to expert readers and QCA. The present decision model assumed that all severe stenoses would trigger referral to ICA and that ICA holds perfect sensitivity and specificity.

## 6 CONCLUSIONS

Application of AI to typically acquired CCTA is a clinically effective, safe and cost savings approach to guide referral management of patients being considered for ICA.

## CONFLICT OF INTEREST STATEMENT

J. P. E. is an employee and retains equity in Cleerly Inc. J. K. M. serves as an employee and reports equity interest in Cleerly Inc.; serves on the Scientific Advisory Board for Arineta; reports equity interest in Upside Foods; and receives research funding from the National Institutes of Health. T. C. is an employee of Cleerly. A. D. C. reports grant funding from G. W. Heart and Vascular Institute, and modest equity in Cleerly and is a consultant for Siemens Healthineers. The remaining authors declare no conflict of interest.

## Data Availability

Data may be obtained from a third party and are not publicly available. Index data for the CONSERVE trial has been previously published. Deidentified patient data are not publicly available, except if necessary to confirm study results; requests for data may be made by contacting Drs. Hyuk‐Jae Chang or James Min.
