# Correction to “Atherosclerosis imaging quantitative computed tomography (AI‐QCT) to guide referral to invasive coronary angiography in the randomized controlled CONSERVE trial”

**DOI:** 10.1002/clc.24083

**Published:** 2023-07-27

Kim Y, Choi AD, Telluri A, et al. Atherosclerosis Imaging Quantitative Computed Tomography (AI‐QCT) to guide referral to invasive coronary angiography in the randomized controlled CONSERVE trial. *Clin Cardiol*. 2023;46:477‐483. doi:10.1002/clc.23995

The affiliations of two authors were incorrect and should have appeared as follows:

Hyung Bok Park, MD, PhD

Division of Cardiology, Department of Internal Medicine, Catholic Kwandong University International St. Mary's Hospital, Incheon, South Korea

Sang‐Eun Lee, MD, PhD

Division of Cardiology, Department of Internal Medicine, College of Medicine, Ewha Womans University, Seoul, South Korea

We apologize for this error.

